# Teleworking: A Curse or a Blessing for Gender Equality and Work-Life Balance?

**DOI:** 10.1007/s10272-021-0995-4

**Published:** 2021-10-05

**Authors:** Manuela Tomei

**Affiliations:** Conditions of Work and Equality Department, ILO, Route des Morillons 4, 1211 Geneva 22, Switzerland

## Abstract

Affordable, reliable and high-quality child and elderly care services are essential for employees to do teleworking in an efficient manner.

The COVID-19 pandemic has fast-tracked the adoption of teleworking modalities by employers everywhere. Teleworking has permitted many companies and organisations to keep functioning, while shielding employees from exposure to the virus during nationwide lockdowns and other containment measures introduced by governments across the globe. As some parts of the world are transitioning to a form of normalcy, working from home seems to be destined to become a more common feature of corporate culture in the post-COVID-19 era, too. In the past months, a number of big companies have announced that they would allow employees to telework in a "hybrid format", combining in-presence work with working from home. Governments have not remained idle either, with many of them adopting new laws in this domain or considering doing so. In such a context, it is legitimate to ask whether the wider adoption of teleworking might narrow the gender divide and help dismantle a corporate culture of "presenteeism" that penalises women, or if it will make gender inequalities worse. Who is more likely to take up teleworking in the future, women or men? What consequences will it have for their careers, training opportunities, remuneration and the gender pay gap? What will be the implications for gender equality at home? This article explores these issues and concludes that policies, both at the national and workplace level, matter. Depending on their design and implementation, they can help respond to workers' demands for enhanced flexibility, while challenging the unequal gender division of work at home. This would not only translate into greater gender equality but would also improve the well-being and productivity of all workers.

* The views expressed herein are those of the author and do not necessarily reflect the views of the International Labour Organization.

**Manuela Tomei**, International Labour Organization, Geneva, Switzerland.

## Teleworking: Opportunities and risks

Before the pandemic, a fraction of the workforce was teleworking occasionally, namely, working from home or a location outside of the employer's premises. Within the EU, the incidence of regular teleworking ranged from 30% in Denmark and the Netherlands to 10% or less in Greece and Italy. In the United States, remote work was performed occasionally by up to 20% of the workforce, while in Japan and Argentina this percentage was smaller, at 16% and 1.6%, respectively (Eurofound and ILO, 2017). Since the outbreak of the pandemic, teleworking saw a spectacular increase, with growth being higher in those countries where teleworking was more common before the health crisis. For instance, in Finland, 60% of employees shifted to working from home, while in Italy and Austria 40% of employees switched to this form of work (Eurofound, 2020).

Further, the empirical evidence regarding the effect of the wider use of teleworking on productivity is inconclusive, with some studies reporting a drop in productivity and others observing short-term productivity gains (OECD, 2020; CIPD, 2020). While further research is clearly needed to draw any definitive conclusions, positive results in some instances have helped challenge the entrenched view that workers who are "out of sight" are less performant, thereby making teleworking a more attractive option for many big companies and organisations. Workers' greater flexibility in deciding when, where and how to work should make it easier for them to accommodate their family life and other commitments and interests. This, in turn, is conducive to greater job satisfaction, creativity and innovation, which are all key to achieving productivity gains. The money and time not spent on commuting provides workers with additional time that they can devote to family or housework, sports or leisure activities. A reduction in the number of daily commutes also benefits the environment through a decline in $CO_2$ emissions and traffic congestion. For large organisations and businesses, relying on employees who split their time between home and the office means lower fixed costs.

But teleworking also carries a number of risks and disadvantages, and the pandemic, with governments and businesses mandating or encouraging teleworking on a full-time basis, has further exposed them. Challenges include, among others, long working hours, insufficient rest breaks and conflict between work and family life. Some surveys show that workers use a considerable portion of the time saved by not commuting to advance or complete work. For example, a study by researchers at Stanford University and the University of Chicago found that Americans devoted 35% of their time normally used for commuting to their primary job (Barrero et al., 2020). Other research in Israel reported that workers performing telework would work 47 minutes longer per day (The Economist, 2020). While this is the case for men and women, the latter tend to devote a larger portion of the time saved by not commuting to household chores or child minding than men (The Economist, 2020; Lyttelton et al., 2020).

Heavy workloads, organisations' and companies' expectations regarding constant availability, alongside self-imposed work intensity – the so-called autonomy paradox – are among the underlying causes. During the current crisis, the limitations of management by control and its inadequacy to the reality of a virtual workplace have become more apparent. Supervisors' requests for employees to provide constant reports on progress made, the establishment of unrealistic targets or the convening of unnecessary meetings at inappropriate times are a reflection of the ingrained belief that employees who work outside the employer's premises feel less pressured to perform. These practices often mirror a mistrust in people's commitment and capacity to work in the absence of direct oversight. The use, in some instances, of intrusive surveillance systems to monitor employees' performance also raises serious concerns regarding the respect of workers' privacy and dignity. While organisations have a legitimate need to monitor workers' performance and protect companies' assets, this need cannot be pursued at the expense of workers' right to privacy.

All of the above point to the need for a change in traditional management styles and methods. Overseeing a dispersed workforce through virtual means calls for a shift from "management by control" to "management by results". The latter rests on setting clear and more realistic expectations; re-assessing performance criteria – for example, by not frowning on employees for working outside core hours; providing adequate means, including equipment and training, for workers to work efficiently from home; determining normal hours of work, availability and rest; devising sensible reporting and feedback mechanisms; and facilitating co-worker networking (ILO, 2020).

A related issue, which has taken centre stage in national and international policy debates, is whether there should be a right to disconnect and which would be the best way of ensuring its implementation, whether through regulation, collective bargaining, company-level policies or non-binding practical guidance. It is clear that implementation would very much depend on national practices and the prevalence of social dialogue. However, regardless of the approach pursued, a number of good practices are worth considering. First, the importance of clarifying why the right to disconnect is necessary and what it entails, including the duty of others to respect this right by, for instance, not sending e-mails outside working hours. Second, making it clear that the employer has an obligation to safeguard the right to disconnect, and that the onus to enforce it does not fall solely on the worker's shoulders.

Third, giving employees certainty concerning when they can switch off, which presupposes defining the hours of rest, normal hours of work and periods of availability, as well as information on how working time is recorded. Fourth, raising awareness on relevant legislation or collective agreements, and supplying clear information to employees at all levels regarding the purpose, content and method of implementation of the related company policy.

## The gender dimensions of teleworking: Before and during the pandemic

The right to disconnect is an important means to draw a boundary between hours of work and non-work, prevent anxiety and burnout, allow for work-life balance and foster family co-responsibility (Eurofound, 2020). A proclaimed objective of teleworking is to support working parents, especially mothers, and reduce gender inequalities in employment (Eurofound and ILO, 2017). While teleworking may make it easier for mothers to juggle paid work and family responsibilities, empirical evidence shows that they do a disproportionate amount of housework and childcare compared to fathers. This suggests that teleworking may help address childcare issues, but does not necessarily foster co-responsibility between fathers and mothers or create more gender equal workplaces unless there is a conscious decision to do so (Social Europe, 2020; Eurofound, 2021).

It is not surprising therefore that in pre-COVID-19 times, women tended to perform teleworking on a more regular basis than men did. Moreover, while both women and men were appreciative of the advantages of teleworking, women were more likely to report feelings of professional and social isolation. Working from home carries a risk of partial "invisibility", and fewer opportunities to interact, learn, cooperate and innovate, with adverse consequences for pay, promotions and training opportunities. This, in turn, reinforces the employers' perception that fathers are more reliable and in need of work more than mothers, because they are the ones who put food on the table and provide shelter for the family. This translates into preferential treatment of fathers over mothers in terms of pay and careers, known as the "fatherhood premium" and the "motherhood penalty", which intensifies over the years (ILO, 2018).

There was some hope that men's protracted use of teleworking during the COVID-19 pandemic would have two important effects on both them and companies. The hope was that men would become more aware of the importance and joy of greater involvement in the upbringing of their children and, hence, more willing to take on a larger share of care and housework. At the same time, employers would realise that employees' commitment and performance are not contingent on their continued presence in the workplace and, thus, become less prejudiced towards employees taking up regular teleworking. These two effects combined would increase the probability that teleworking may deliver better on gender equality at home and the workplace.

Did the pandemic and mandatory teleworking help alter the skewed distribution of unpaid care work towards women? There has been some progress, but still too modest. During the lockdown and other confinement measures, the number of meals to be prepared per day as well as the time devoted to home schooling or looking after family members with COVID-19 increased significantly. Men took on part of these additional tasks, but women absorbed the brunt of them.

Within the EU, women, especially women with children below the age of 12, reported problems in concentrating on their job all or most of the time and have had a particularly hard time in meeting the expectations of their employers and coping with the additional housework and family responsibilities (Mascherini, 2020). A survey in the UK shows that during the lockdown mothers reported being interrupted by their children 50% more frequently than fathers (Andrew et al., 2020). In Turkey, men's participation in unpaid work increased, particularly for men who switched to working from home, but the relative increase for women further widened the gender gap in unpaid work (İlkkaracan and Memiş, 2021). Single mothers with young children find it particularly challenging to meet tight deadlines or attend meetings at times in which they have to feed their children or help them with their homework (Eurofound, 2020a; ILO, 2020a).

Many more women than men have dealt with this extraordinary pressure by reducing their hours of work, with the consequent loss in their current and, most likely, future earnings, by taking parental leave or by quitting their job and the labour market altogether. In North America, one in four female employees, including senior level employees, said they would stop working or reduce the number of hours because of company inflexibility, caring responsibilities and stress (McKinsey and LeanIn, 2020). Supporting the partner who makes more money in the couple is another reason (Romei, 2021). Compared to men, women appear to be more likely to experience psychological stress and anxiety due to the uncertainties brought about by the pandemic and the loss of relatives and friends. Further, domestic violence against women has worsened during the pandemic (ILO, 2020b; EU, 2021).

A number of countries extended care support and introduced special parental leave schemes as part of their efforts to protect working parents against job and income losses. In some countries, however, eligibility to special parental leave schemes ruled out those parents who could work from home, reflecting not only a gross underestimation of time demands on working parents in the midst of a pandemic, but also the view that teleworking is a solution to childcare problems (Rubery and Tavora, 2021). Teleworking performed during the trying circumstances of the pandemic is not comparable with teleworking carried out in "normal" times. However, the experience of these past 18 months has made it even clearer that, as long as teleworking will not be organised in ways that will challenge the assumption that home is where women belong and the office is where men belong, gender imbalances will continue unabated at home and work.

Recent surveys conducted in the US, UK, EU and Vietnam (Nguyen and Armoogum, 2021) asking workers whether they would like to continue teleworking at least one or more days a week, confirm that more women than men prefer doing so, although the percentage of men answering positively to the question is larger than in the pre-COVID-19 era. Women's greater preference for teleworking relative to men does not come as a surprise since, in the absence of supportive social policies and infrastructure, teleworking allows them to make a living, while looking after their homes. Men's increased willingness to work remotely on a regular basis is certainly encouraging and suggests that the pandemic has had some effect in modifying their views regarding their role at home. It also suggests that the pandemic has somehow contributed to reducing the stigma associated with working from home, as reflected in the announcement of a number of big companies to make teleworking available, at least for some of their employees, in a hybrid format (The Economist, 2020a, 2021).

However, we cannot rule out that more men than women will return to work in presence more days a week, especially if they are the higher income earners (Hickok, 2021). If this proves to be the case, women's career prospects would take a hit and the "fatherhood premium" and "motherhood penalty" would gain further steam. This is why the European Economic and Social Committee (EESC), under the Portuguese Presidency of the European Commission, has issued an opinion, the purpose of which is to prevent teleworking from further widening the unequal division of work at home between women and men (EESC, 2021). In addition, the G20 countries under the Italian Presidency have adopted a Ministerial Declaration and roadmap on teleworking that addresses this very concern and puts forward a number of policy recommendations (G20, 2021).

## Conclusions

No single policy or actor will make teleworking an engine to counter the rise in gender imbalances during the pandemic, but rather a combination of public and workplace policies and changes in the behaviour of workers.

It is clear that more men need to take up flexible working arrangements. To this end, companies should encourage their use across the board – from seniority to entry level – and not leave the decision of whether and how frequently to work remotely exclusively to staff. For example, a policy requesting that all employees take an equal number of work-from-home days could be a possibility. This would reduce the risk that women with caring duties end up staying at home more often than the remaining staff. A careful monitoring of who takes up the option of working from home, once coronavirus restrictions are lifted, and with which results, would permit prompt corrective action to be taken, if and as needed.

For greater uptake of working remotely, employees who do teleworking should not be penalised relative to those who work at the office. This does not only mean that human resources management policies should envisage equal treatment and opportunities between remote and office workers in terms of pay, career advancement or training opportunities, including regarding digital skills. Management should also contribute to the promotion of trust and cooperation between remote and office workers.

Work-home boundary management support is also key to prevent burnout and allow for work-life balance. In addition to informing employees of the normal hours of work, hours of rest and hours of "contactability", which are broader than normal hours, companies can also set "core working hours" to provide further flexibility. The focus, in this case, is on the number of hours worked instead of when they are worked. In addition, shutting down computer servers at night or discouraging managers or peers from contacting employees over weekends could also be helpful, besides providing training on tailor-made individual boundary management strategies.

Affordable, reliable and high-quality child and elderly care services are essential for employees to do teleworking in an efficient manner. This would permit employees working remotely to concentrate on their jobs without constant interruptions.

Leave policies, including parental leave, must not reproduce or reinforce the unequal division of unpaid care work. This means that they should avoid creating disincentives or, rather, should generate incentives for both mothers and fathers to take up these leaves.

Digital connectivity is a key enabling condition for teleworking to function at its best. This requires serious investments to narrow the current digital inequality between countries of different income levels, between urban and rural areas and between women and men.

In sum, while the pandemic has hit women harder than men, and the risk of the reversal of women's hard-won gains is real, the pandemic has also opened up new opportunities to correct structural gender inequalities. Teleworking, if adequately organised, may be one such opportunity. Its widespread use during the pandemic has further exposed its benefits and liabilities, including with respect to gender equality at work and at home. We are clearer in terms of what action is needed and by whom. This crisis should not be wasted.
